# Normative data for handgrip strength in children and adolescents in the Maule Region, Chile: Evaluation based on chronological and biological age

**DOI:** 10.1371/journal.pone.0201033

**Published:** 2018-08-09

**Authors:** Rossana Gómez-Campos, Cynthia Lee Andruske, Miguel de Arruda, Jose Sulla-Torres, Jaime Pacheco-Carrillo, Camilo Urra-Albornoz, Marco Cossio-Bolaños

**Affiliations:** 1 Universidad Autónoma de Chile, Talca, Chile; 2 Faculty of Physical Education, State University of Campinas, Sao Paulo, Brazil; 3 Universidad Nacional de San Agustín, Arequipa, Perú; 4 Centro de Investigación Especializado en Ciencias de la Educación, Salud y Deporte, CINEMAROS, Arequipa, Perú; 5 Universidad Católica de Santa María, Arequipa, Perú; 6 Universidad del Bio Bio, Chillán, Chile; 7 Escuela de Kinesiología, Facultad de Salud, Universidad Santo Tomás, Talca, Chile; 8 Departamento de Ciencias de la Actividad Física, Universidad Católica del Maule, Talca, Chile; Universidad del Desarrollo, CHILE

## Abstract

**Background:**

Hand grip strength (HGS) is associated with a number of causes resulting in cardiovascular death, in addition to bone fragility, and the presence of sarcopenia. The goal of our study was to analyze HGS of students based on chronological and biological age and propose normative standards for children and adolescents from Chile.

**Methods:**

We studied 4604 school children of both sexes between the ages of 6.0 and 17.9 years of age. Weight, standing height, sitting height, and hand grip strength (HGS- right and left) were measured. The Body Mass Index (BMI) was calculated, and the biological age was calculated by using age at peak height velocity (APHV).

**Results:**

When arranged by chronological age, no significant differences occurred in HGS between both sexes of school children from age 6 to 12 years of age. However, from ages 13 to 17, males showed greater HGS than females. Significant differences also emerged between both sexes and at all levels for biological age (APHV). For males, chronological age explained the HGS occurring between 0.74 to 0.75% and for females between 0.54 to 0.59%. For males, biological age explained the HGS for the range of 0.79 to 0.80% and 0.62 to 0.67% for females. The normative data for HGS for both sexes is expressed in percentiles.

**Conclusions:**

HGS during childhood and adolescence needs be analyzed and interpreted in terms of biological age rather than chronological age. The normative data to evaluate the HGS are a tool that can help professionals working in clinical and epidemiological contexts.

## Introduction

Hand grip strength (HGS) is used as a means to predict health throughout an individual’s lifetime [1,2]. It is one of the field tests used the most to measure maximum isometric strength of the grip strength of both of the hands [3]. HGS is an important indicator that helps identify the level of development and degree of disability. It also assists in determining the effectiveness of rehabilitation and evaluation of the integrity of the functioning of the upper extremities [4].

HGS is associated with a number of causes resulting in cardiovascular death [2,5] in addition to bone fragility, and the presence of sarcopenia [6]. Furthermore, low levels of strength are also associated with high mortality rates for individuals developing different types of significant illnesses [5].

In general, hand dynamometry has advantages such as its predictive value, simplicity and ease of the measurement procedure, portability, and low cost [7]. These characteristics appeal to health professionals for evaluating isometric strength in children, adolescents, and adults in clinical and epidemiological settings.

As a result, a growing number of research studies are appearing in the literature that propose using normative data to evaluate HGS during different stages of life and diverse regions of the world [3,8–12]. In general, standards are developed based on chronological age. At the same time, it is widely accepted that the variation between individuals of the same chronological age during puberty is great [13,14]. However, to our knowledge, no research exists based on normative data to evaluate HGS using biological age.

In summary, studying HGS in terms of chronological and biological age could provide relevant information. It could also help prevent confounding effects between and within individuals once chronological intensity and duration during puberty are identified for each adolescent since considerable variation could occur between individuals [15]. Furthermore, the use of foreign data standards is not appropriate since these do not take into account the differences in physical characteristics, race, and ethnic origin among the regions [16].

The hypothesis for this study is that HGS arranged by chronological age could be confounded by biological maturation since females mature approximately two years before males [15,17]. In this sense, the results of this study could help develop references for both chronologic and biological ages.

Therefore, the objectives for this study were the following: a) analyze HGS by chronological age, b) analyze HGS by biological age, and c) propose reference standards for children and adolescents from the Maule Region of Chile.

## Methodology

### Research design and sample

A cross-sectional study was conducted with a sample of 4604 students (2269 males and 2235 females) of both sexes. Students’ ages ranged from 6.0 to 17.9 years. Subjects were invited to participate in the evaluation and measurement process in April of 2015. Data collection was carried out between August and November 2015. Students recruited for this study were selected from 12 public government funded schools from the Maule Region of Chile. Generally, in Chile, students attending public schools are from the middle class [18].

Consent forms to authorize the assessments were sent home to parents through their children. Parents and/or guardians granting permission for their children to participate were visited at the respective schools by the principal researchers for the project.

Due to the low initial acceptance rate by parent and/or guardians, this process was carried out three more times. Finally, from a total of 5345 potential subjects invited, 94 smokers and 647 not receiving parental consent for the measurement process were excluded. Therefore, the sample size consisted of a total of 4604 students. The experimental protocol was based on the Helsinki Declaration (World Medical Association for Humans). Furthermore, this study received approval from the respective school authorities as well as the Ethics Committee from the Universidad Autónoma of Chile (protocol no. 238/2013).

### Data collection procedures

The ages of the subjects studied were collected from students’ registration records. This information was provided by the school administrations. The ages for both sexes were grouped in 10 age categories from 6.0 to 17.9 years. Intervals consisted of one year, for example 6.0 to 6.9 years.

Body weight (kg) was measured using an electric scale (Tanita, United Kingdom, Ltd.) with a scale of 0 to 10 kg and with an accuracy of 100 g. Standing height was measured with a portable stadiometer (Seca & Co. KG, Hamburg, Germany) with a precision of 0.1 mm and a scale of 0–2.50 m. Sitting height was taken using a wooden bench (50 cm high) with a measurement scale of 0 to 150 cm with a 1 mm accuracy. All anthropometric variables were evaluated according to the protocol described by Ross and Marfell-Jones [19]. Body Mass Index (BMI) was calculated by using the formula proposed by Quetelet (BMI = body mass (kg) /standing height (m)^2^).

The evaluation process was carried out by a team of professionals (4 physical education professors) with extensive experience in anthropometric measurement. Ten percent of the sample (n = 464) was measured twice in order to ensure quality of measures. The technical error of measurement (TEM) values were less than 2% for all anthropometric variables.

HGS was measured with the help of a JAMAR (brand) hydraulic dynamometer (Hydraulic Hand Dynamometer® Model PC-5030 J1, Fred Sammons, Inc., Burr Ridge, IL: USA). It has an accuracy of 0.1 lbf for both the right and left hands when following the recommended protocol proposed by Richards et al. [20]. Each subject was seated in a standard position in a chair with a straight back. Students were asked to exert pressure on the dynamometer twice with each hand. To control for effects of fatigue, the attempts were performed by alternating the hands with approximately 2 minutes of rest between each attempt for each hand. The better measurement was recorded for each of the two attempts. The Technical Error of Measurement (TEM) for intra- and inter-evaluator oscillated from 1.2 to 1.8% for both hands.

Biological maturation was determined by using the technique proposed by Mirwald et al. [21]. This is an indicator of somatic maturation that represents the maximum growth period in height during adolescence. To estimate age at peak height velocity (APHV), multiple regression equations by gender were used. Standing height, sitting height, leg length (standing height–sitting height), decimal age, and their interactions were also included. Biological age was created based on one year intervals represented as -6 to 2 APHV in males and for females, -5 to 7 APHV.

### Statistical analysis

The normality of the data was verified through the Kolmogorov-Smirnov test. Descriptive statistics were used to calculate the arithmetic mean and standard deviation. The t-test for independent samples was used to determine the differences between both sexes. To determine the relationship between the variables for chronological and biological age with HGS simple linear regression was calculated (Pearson coefficient and R^2^). The distribution of smoothed percentiles was created for HGS for each sex using the LMS method [22]. LMS Chartmaker Pro version 2.3 [23] software was used to create the percentiles. In addition, a power transformation of Box-Cox was performed for the HGS variable of both hands by chronological and biological age. The maximum penalty probability procedure was implemented in order to create the following smoothed curves: L(t) Box-Cox Power, M(t) median, and S(t) coefficient of variation. The following percentiles were calculated: p3, p10, p15, p25, p50, p75, p85, p90, and p97. For all of the cases, the significance was less than 1%. Analysis was carried out using SPSS 16.0.

## Results

The anthropometric variables of body mass, standing height, and body mass index of both sexes are illustrated in Table 1 below. Values for body mass are similar in both sexes from age 6 to 13 years. From age 14 to 17 years, males showed greater weight when compared to females (p<0.001). With regards to standing height, no differences were found between both sexes ages 6, 7, 8, and 12 years. At ages 9, 10, and 11 years old, females had grown taller than the males (p<0.001), and from age 13 to 17 years old, males showed significantly higher values in height than the females (p<0.001). With regard to sitting height, no significant differences occurred at ages 6, 7, 8, 9, and 13 years. However, at 10, 11, and 12 years old, females showed higher values in sitting height (p<0.001). Subsequently, from 14 to 17 years old, males showed higher values than their counterparts (p<0.001).

**Table 1:** Anthropometric characteristics of the sample studied.

Age (years)	n	Body weight (kg)		Standing height (cm)		Sitting height (cm)		BMI (kg/m^2^)		Biological age (APHV)	
		X	SD	X	SD	X	SD	X	SD	X	SD
Males											
6.0–6.9	133	26.8	7.3	119.6	4.7	64.2	5.3	18.7	4.6	-5.9	0.3*
7.0–7.9	72	30.6	5.9	127.4	5.5	68.1	6.0	18.7	2.7	-5.2	0.5*
8.0–8.9	96	31.6	6.2	130.8	5.6	68.1	3.6	18.4	2.6	-4.8	0.4*
9.0–9.9	154	36.1	6.3	136.6	7.7*	70.9	2.7	19.4	3.4	-4.2	0.3*
10.0–10.9	189	41.0	9.4	141.2	8.1*	72.8	3.9*	20.3	3.0	-3.6	0.4*
11.0–11.9	118	45.5	11.2	147.2	7.4*	75.8	3.2*	20.8	3.9	-2.8	0.4*
12.0–12.9	150	48.5	12.0	153.0	6.9	77.8	4.3*	20.6	4.4*	-2.2	0.5*
13.0–13.9	185	54.6	11.8	160.5	9.3*	82.0	5.3	21.0	3.4	-1.3	0.6*
14.0–14.9	300	61.3	12.1*	166.2	6.8*	84.3	4.7*	22.1	3.7*	-0.5	0.6*
15.0–15.9	200	66.6	9.1*	169.7	7.0*	87.6	3.9*	23.1	3.2*	0.4	0.5*
16.0–16.9	287	70.3	13.6*	170.9	8.5*	89.6	4.4*	24.1	4.5*	1.2	0.7*
17.0–17.9	385	70.6	12.3*	171.4	6.5*	89.3	3.8*	24.0	3.7	1.6	0.6*
Females											
6.0–6.9	161	27.9	17.8	120.3	7.8	64.4	7.0	19.5	3.7	-4.9	0.6
7.0–7.9	124	30.9	7.7	128.8	11.9	68.0	4.7	18.3	3.1	-4.0	0.7
8.0–8.9	101	31.7	7.8	129.7	7.3	68.3	3.6	18.6	3.2	-3.3	0.5
9.0–9.9	243	37.6	8.4	159.7	16.1	71.5	5.0	19.1	4.0	-2.1	0.7
10.0–10.9	269	41.7	8.4	144.5	6.9	75.7	5.4	19.9	3.1	-0.9	0.6
11.0–11.9	165	45.7	9.9	149.3	8.4	77.2	5.2	20.3	3.6	0.1	0.8
12.0–12.9	148	49.5	10.6	154.9	5.4	80.7	4.6	22.2	3.7	1.5	0.8
13.0–13.9	119	54.7	10.9	156.9	9.3	82.4	2.9	22.3	3.9	2.5	0.9
14.0–14.9	271	58.7	11.5	158.8	7.3	82.8	4.5	23.2	3.9	3.6	0.9
15.0–15.9	167	60.9	11.0	159.5	4.0	83.9	3.0	23.9	4.2	4.5	0.7
16.0–16.9	247	63.5	13.4	158.6	7.7	83.5	3.6	25.5	8.0	5.4	1.0
17.0–17.9	359	62.3	12.6	158.3	5.4	83.3	3.2	24.8	4.5	6.1	0.9

X: Mean, SD: Standard deviation, BMI: Body Mass Index

* Significant difference p<0.05.

With respect to BMI, no significant differences were found between both sexes at ages 6, 7, 8, 9, 10, 11, 13, and 17. However, at 12, 14, 15, and 16 years old, females had greater BMI than males (p<0.001).

Biological age (APHV) varied between -5.9 and 1.6 for males, and significant differences occurred in females for all age groups between both sexes.

**Fig 1:**
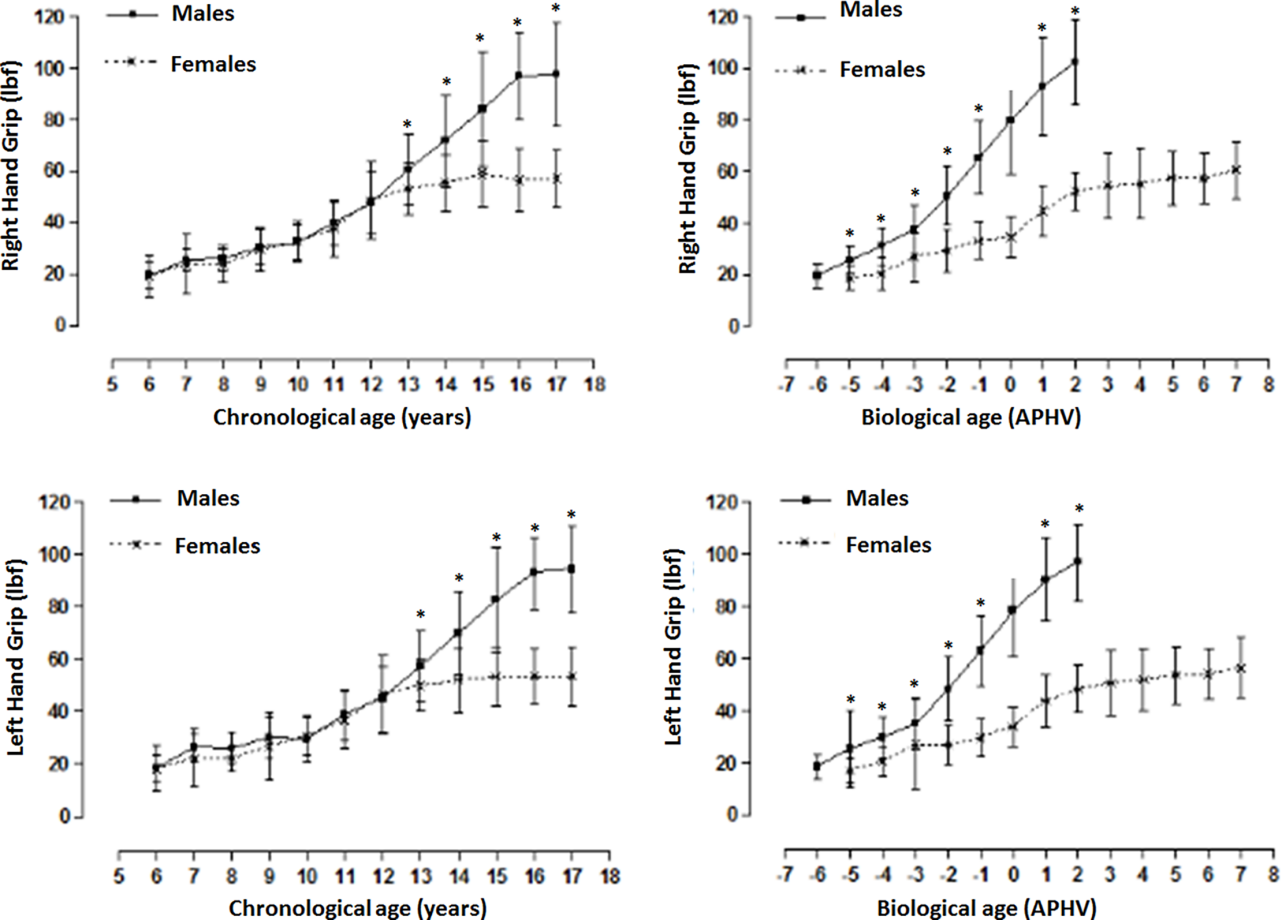
**Handgrip strenght (right and left) arranged by chronological and biological age**.

Fig 1 illustrates the handgrip strength (HGS) of both sexes arranged by chronological and biological age. Note that for both hands, no significant differences occurred between males and females from 6 to 12 years old. However from age 13 to 17 years, males demonstrated greater HGS than did the females (p<0.001). On the other hand, when HGS was arranged by biological age (APHV), significant differences occurred between both sexes. Males showed greater HGS (right and left) from -5APHV until 2APHV in comparison to females (p<0.001).

Simple linear regression analysis (Table 2) results indicated that chronological and biological age (APHV) were related to manual handgrip strength in children and of both sexes. The explanation for the % of variation in both sexes is greater when biological age is analyzed (males R^2^ = 0.79–0.80 and females R^2^ = 0.62–0.67) rather than by chronological age (males R^2^ = 0.74–0.75 and females R^2^ = 0.54–0.59).

**Table 2:** Relationship between HGS (right and left) and chronological and biological age for both sexes.

	Chronological age (years)				Biological age (APHV)			
	R	R^2^	SEE	p	R	R^2^	SEE	p
**Males**								
HGS right (lbf)	0.86	0.74	16.25	0.000	0.88	0.79	14.70	0.000
HGS left (lbf)	0.87	0.75	15.21	0.000	0.89	0.80	13.90	0.000
**Females**								
HGS right (lbf)	0.77	0.59	11.17	0.000	0.82	0.67	10.07	0.000
HGS left (lbf)	0.74	0.54	11.47	0.000	0.78	0.62	10.54	0.000

SEE: Standard error of estimate, APHV: age at peak height velocity.

**Fig 2:**
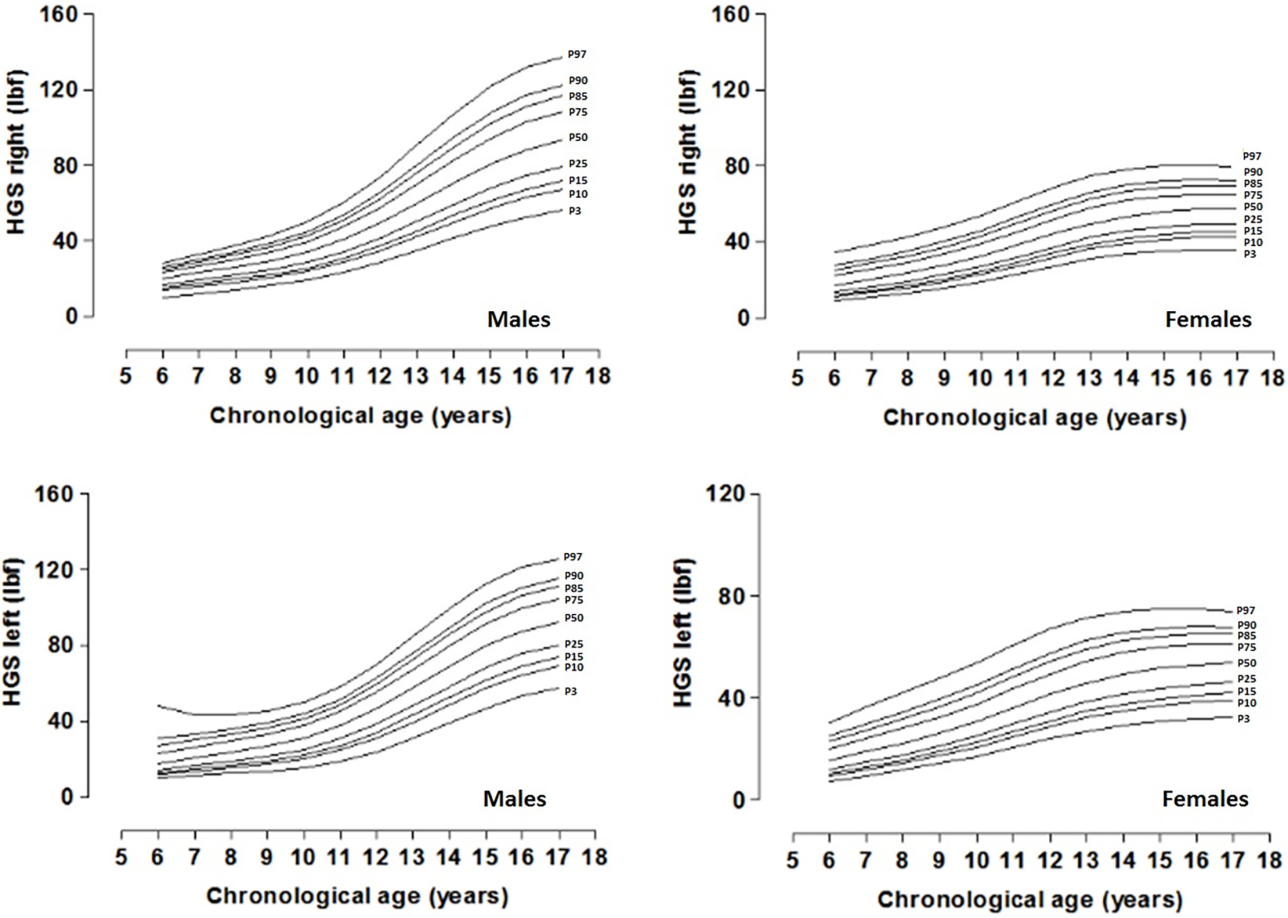
Smoothed centile curves for handgrip strenght (P3, P10, P15, P25, P50, P75, P85, P90, and P97) by chronological age for males and females. The solid lines indicate P3, P10, P15, P25, P50, P75, P85, P90, and P97: Percentiles.

**Fig 3:**
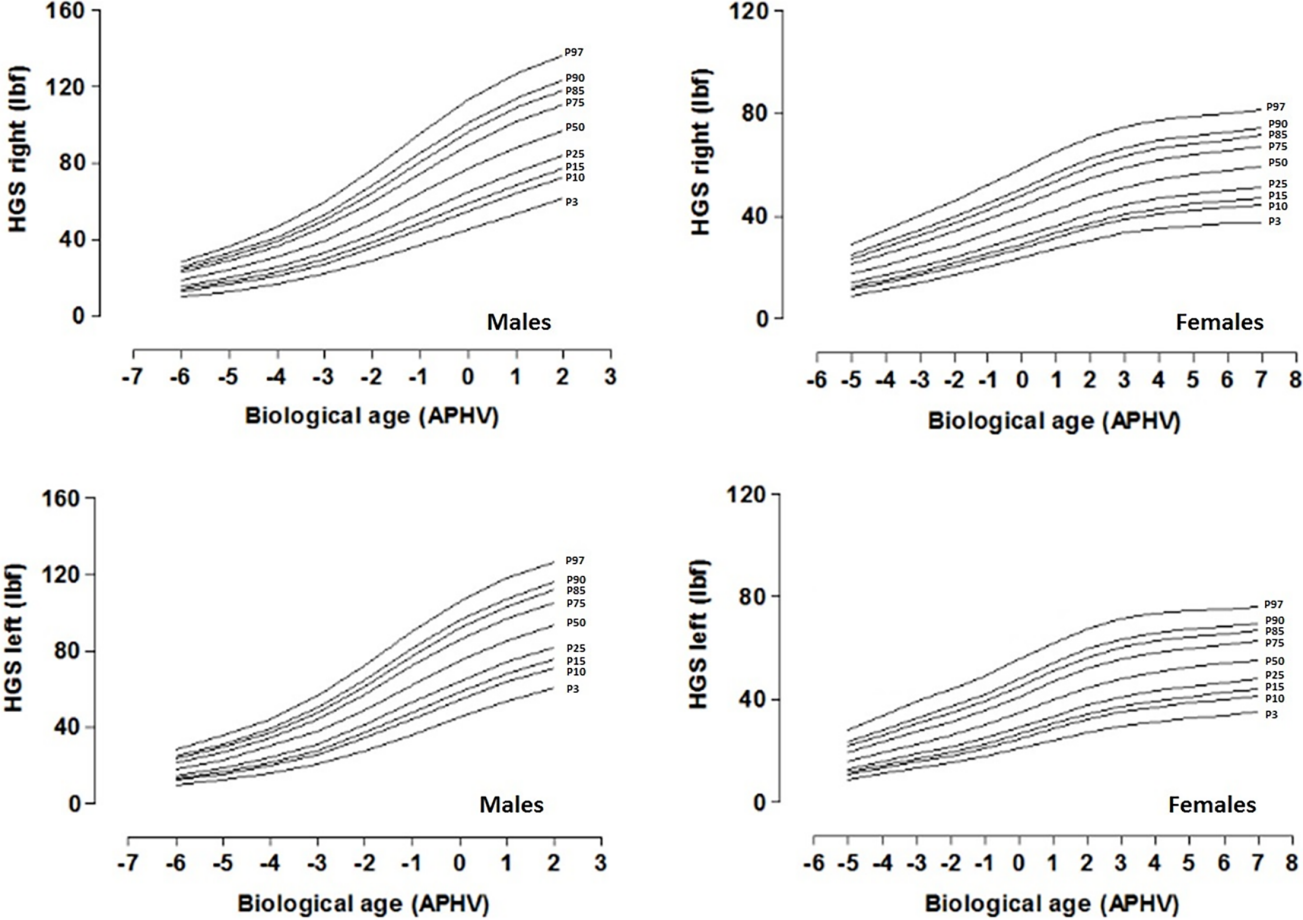
Smoothed centile curves for handgrip strenght (P3, P10, P15, P25, P50, P75, P85, P90, and P97) by biological age for males and females. The solid lines indicate P3, P10, P15, P25, P50, P75, P85, P90, and, P97: Percentiles.

The distribution of percentiles by chronological and biological age is presented in Tables 3 and 4 and Figs 2 and 3. In both cases, the mean values increase as chronological and biological ages advance.

**Table 3:** Percentile values for handgrip strength by sex and chronological age.

Chronological age (years)		HGS right (lbf)												HGS left (lbf)											
	n	L	M	S	P3	P10	P15	P25	P50	P75	P85	P90	P97	L	M	S	P3	P10	P15	P25	P50	P75	P85	P90	P97
**Males**																									
6.0–6.9	133	1.50	20.38	0.23	10.3	13.9	15.2	17.1	20.4	23.4	24.9	26.0	28.4	-0.88	17.36	0.36	10.2	11.8	12.6	13.9	17.4	22.8	27.2	31.2	48.1
7.0–7.9	72	1.32	23.51	0.23	12.4	16.2	17.7	19.8	23.5	27.1	28.9	30.1	33.1	-0.38	20.55	0.34	11.6	13.7	14.7	16.5	20.6	26.2	30.1	33.2	43.0
8.0–8.9	96	1.14	26.42	0.23	14.4	18.4	19.9	22.2	26.4	30.5	32.7	34.1	37.7	0.08	23.63	0.33	12.6	15.4	16.8	18.9	23.6	29.4	33.0	35.6	43.0
9.0–9.9	154	0.99	29.76	0.24	16.6	20.8	22.5	25.0	29.8	34.5	37.1	38.8	43.0	0.47	26.98	0.31	13.6	17.4	19.0	21.6	27.0	32.9	36.4	38.9	45.2
10.0–10.9	189	0.85	34.22	0.24	19.4	24.0	25.9	28.8	34.2	39.8	42.9	45.0	50.1	0.75	31.40	0.29	15.3	20.1	22.2	25.3	31.4	37.8	41.3	43.8	49.9
11.0–11.9	118	0.73	40.77	0.24	23.4	28.6	30.9	34.2	40.8	47.6	51.4	54.0	60.5	0.92	38.02	0.28	18.6	24.7	27.2	30.9	38.0	45.2	49.1	51.7	58.3
12.0–12.9	150	0.63	49.51	0.25	28.6	34.8	37.5	41.6	49.5	58.0	62.7	66.0	74.3	1.00	46.99	0.26	23.8	31.2	34.2	38.7	47.0	55.3	59.7	62.8	70.1
13.0–13.9	185	0.55	59.86	0.25	34.9	42.2	45.4	50.2	59.9	70.2	76.1	80.2	90.6	1.02	57.86	0.25	30.9	39.5	43.1	48.2	57.9	67.4	72.6	76.0	84.5
14.0–14.9	300	0.51	70.64	0.25	41.6	50.0	53.7	59.3	70.6	82.9	89.9	94.8	107.3	1.02	69.44	0.23	39.1	48.8	52.8	58.6	69.4	80.2	86.0	89.9	99.5
15.0–15.9	200	0.50	80.48	0.24	47.7	57.2	61.4	67.8	80.5	94.3	102.1	107.6	121.7	1.03	79.96	0.22	47.1	57.6	61.9	68.2	80.0	91.7	97.9	102.1	112.5
16.0–16.9	287	0.54	88.28	0.24	52.8	63.2	67.7	74.6	88.3	103.0	111.3	117.1	131.8	1.06	87.81	0.21	53.5	64.5	69.0	75.6	87.8	99.9	106.4	110.7	121.4
17.0–17.9	385	0.62	93.68	0.23	56.6	67.7	72.4	79.6	93.7	108.6	117.0	122.8	137.5	1.12	92.53	0.20	57.5	68.9	73.5	80.2	92.5	104.6	111.1	115.4	125.9
**Females**																									
6.0–6.9	161	-0.17	17.57	0.35	9.5	11.4	12.4	14.0	17.6	22.3	25.5	27.9	35.1	0.22	15.70	0.38	7.2	9.4	10.4	12.1	15.7	20.1	22.9	24.9	30.5
7.0–7.9	124	-0.10	20.65	0.33	11.4	13.7	14.8	16.6	20.6	25.8	29.1	31.7	38.9	0.07	18.87	0.36	9.5	11.8	12.9	14.8	18.9	24.0	27.2	29.7	36.5
8.0–8.9	101	-0.03	23.99	0.31	13.5	16.2	17.5	19.5	24.0	29.5	33.0	35.7	43.0	-0.02	22.25	0.34	11.8	14.4	15.7	17.7	22.2	28.0	31.6	34.4	42.2
9.0–9.9	243	0.03	28.01	0.29	16.2	19.3	20.8	23.1	28.0	34.0	37.7	40.5	48.0	-0.02	26.14	0.32	14.4	17.4	18.8	21.1	26.1	32.4	36.4	39.4	47.9
10.0–10.9	269	0.08	32.86	0.27	19.5	23.1	24.7	27.3	32.9	39.4	43.4	46.3	54.2	0.05	30.80	0.30	17.3	20.8	22.5	25.1	30.8	37.7	42.0	45.2	53.9
11.0–11.9	165	0.13	38.56	0.26	23.4	27.6	29.4	32.4	38.6	45.7	50.1	53.2	61.5	0.15	36.07	0.29	20.6	24.7	26.6	29.7	36.1	43.6	48.2	51.5	60.5
12.0–12.9	148	0.20	44.58	0.24	27.6	32.3	34.4	37.7	44.6	52.4	57.0	60.3	69.0	0.25	41.36	0.27	24.0	28.8	30.9	34.3	41.4	49.5	54.3	57.7	66.9
13.0–13.9	119	0.31	49.85	0.23	31.3	36.5	38.9	42.5	49.8	58.1	62.8	66.2	75.0	0.39	45.92	0.26	26.9	32.3	34.7	38.4	45.9	54.3	59.2	62.6	71.5
14.0–14.9	271	0.44	53.77	0.22	34.0	39.7	42.2	46.1	53.8	62.1	66.9	70.3	78.8	0.57	49.38	0.24	29.1	35.0	37.6	41.6	49.4	57.8	62.5	65.8	74.1
15.0–15.9	167	0.59	56.15	0.21	35.5	41.7	44.3	48.3	56.2	64.5	69.2	72.4	80.6	0.76	51.66	0.23	30.6	37.0	39.7	43.8	51.7	59.8	64.3	67.4	75.1
16.0–16.9	247	0.75	57.19	0.21	36.0	42.5	45.2	49.3	57.2	65.3	69.8	72.9	80.5	0.95	53.02	0.22	31.6	38.3	41.1	45.3	53.0	60.8	65.0	67.9	74.9
17.0–17.9	359	0.92	57.37	0.20	35.9	42.6	45.4	49.6	57.4	65.3	69.5	72,4	79.6	1.13	53.77	0.21	32.4	39.3	42,1	46.2	53.8	61.2	65.1	67.7	74.1

L: lambda, M: mean, and S: sigma.

**Table 4:** Percentile values for handgrip strength by sex and biological age.

Biological age (APHV)		HGS right (lbf)												HGS left (lbf)											
	n	L	M	S	P3	P10	P15	P25	P50	P75	P85	P90	P97	L	M	S	P3	P10	P15	P25	P50	P75	P85	P90	P97
**Males**																									
-6	134	0.93	19.2	0.26	9.9	12.8	14.0	15.8	19.2	22.7	24.5	25.8	28.	0.56	17.9	0.28	9.6	12.0	13.1	14.7	17.9	21.5	23.5	24.9	28.5
-5	147	0.86	24.4	0.26	13.0	16.5	18.0	20.2	24.4	28.8	31.1	32.8	36.7	0.67	22.8	0.27	12.2	15.3	16.7	18.8	22.8	27.1	29.6	31.2	35.5
-4	282	0.78	30.9	0.26	16.9	21.1	22.9	25.6	30.9	36.3	39.3	41.3	46.4	0.76	29.1	0.27	15.5	19.6	21.4	24.0	9.,1	34.4	37.3	39.3	44.3
-3	241	0.71	39.5	0.25	22.1	27.3	29.5	32.9	39.5	46.4	50.2	52.9	59.5	0.83	37.5	0.26	20.3	25.6	27.8	31.2	37.5	44.1	47.7	50.1	56.2
-2	184	0.66	50.9	0.25	29.1	35.6	38.4	42.6	50.9	59.7	64.7	68.1	76.7	0.86	48.9	0.24	27.4	34.1	36.8	41.0	49.0	57.1	61.6	64.6	72.1
-1	263	0.63	64.1	0.24	37.3	45.2	48.6	53.9	64.1	74.9	81.0	85.2	95.9	0.86	62.2	0.23	36.1	44.2	47.6	52.6	62.2	71.9	77.2	80.9	89.9
0	302	0.66	76.9	0.24	45.6	55.0	59.0	65.1	77.0	89.5	96.5	101.3	113.5	0.89	74.9	0.22	45.3	54.6	58.4	64.1	75.0	86.0	91.9	96.0	106.0
1	425	0.72	88.1	0.22	53.7	64.1	68.5	75.2	88.1	101.5	109.0	114.1	126.9	0.96	85.6	0.20	53.5	63.7	67.8	74.0	85.6	97.2	103.5	107.7	118.2
2	291	0.78	97.1	0.2	61.4	72.4	77.0	83.9	97.1	110.7	118.2	123.3	136.0	1.07	93.8	0.19	60.3	71.1	75.5	81.9	93.8	105.6	111.9	116.2	126.5
**Females**																									
-5	155	0.52	17.7	0.30	9.0	11.5	12.6	14.2	17.7	21.4	23.6	25.1	29.0	0.06	15.9	0.31	8.8	10.7	11.6	12.9	15.9	19.6	21.9	23.5	28.1
-4	118	0.36	21.1	0.29	11.5	14.2	15.4	17.3	21.1	25.5	28.1	29.9	34.7	-0.02	19.3	0.30	11.1	13.2	14.2	15.8	19.3	23.6	26.3	28.3	33.9
-3	135	0.22	24.8	0.28	14.3	17.2	18.5	20.5	24.8	29.8	32.7	34.9	40.5	-0.06	22.6	0.29	13.3	15.7	16.8	18.7	22.6	27.5	30.6	32.9	39.3
-2	201	0.13	28.7	0.26	17.3	20.4	21.8	24.0	28.7	34.2	37.5	39.9	46.3	-0.03	25.9	0.28	15.4	18.2	19.5	21.5	26.0	31.3	34.7	37.2	44.0
-1	237	0.08	32.9	0.25	20.4	23.8	25.3	27.8	32.9	38.9	42.5	45.1	52.1	0.05	29.9	0.27	17.9	21.1	22.6	24.9	29.9	35.8	39.4	42.0	49.2
0	129	0.08	37.6	0.24	23.8	27.6	29.3	32.0	37.6	44.1	48.0	50.8	58.3	0.14	34.7	0.26	20.9	24.7	26.4	29.0	34.7	41.2	45.1	47.9	55.5
1	144	0.12	42.6	0.23	27.4	31.6	33.5	36.5	42.6	49.6	53.8	56.8	64.8	0.24	39.8	0.25	24.2	28.6	30.5	33.5	39.8	46.9	51.2	54.2	62.1
2	118	0.19	47.4	0.22	30.8	35.5	37.5	40.8	47.4	54.8	59.2	62.3	70.5	0.34	44.5	0.24	27.2	32.1	34.3	37.6	44.5	52.0	56.4	59.6	67.6
3	202	0.30	51.3	0.21	33.5	38.6	40.8	44.3	51.3	59.0	63.5	66.6	74.8	0.47	48.1	0.23	29.6	35.0	37.3	40.9	48.1	55.9	60.4	63.5	71.5
4	259	0.45	54.1	0.21	35.3	40.8	43.2	46.8	54.1	61.9	66.4	69.5	77.4	0.61	50.7	0.22	31.3	37.0	39.5	43.3	50.7	58.5	62.9	65.9	73.5
5	277	0.62	56.1	0.20	36.4	42.3	44.8	48.6	56.1	63.9	68.3	71.3	78.9	0.74	52.5	0.21	32.6	38.6	41.2	45.1	52.5	60.1	64.4	67.3	74.5
6	239	0.79	57.7	0.20	37.0	43.4	46.0	50.0	57.7	65.5	69.8	72.7	80.0	0.87	53.9	0.20	33.8	40.0	42.7	46.5	53.9	61.4	65.5	68.3	75.1
7	121	0.97	59.4	0.20	37.3	44.3	47.2	51.4	59.4	67.3	71.6	74.5	81.7	0.99	55.4	0.20	35.0	41.5	44.1	48.1	55.4	62.7	66.7	69.3	75.9

L: Lambda, M: Mean and S: Sigma, biological age was calculated in years to estimate age at peak height velocity (APHV).

## Discussion

With regard to the analysis of the HGS by chronological age in this study, the results showed similar values from ages 6 to 12 years old. Subsequently, males demonstrated greater HGS than their female counterparts. These findings are similar to those of other international studies that describe differences during the period of adolescence [10,11,24]. However, when the results were arranged by biological age, males demonstrated greater HGS when compared to the females at all levels of the APHV.

Therefore, these findings demonstrate that the utility of chronological age is limited for analyzing HGS during the growth and biological maturation processes. Thus, the range of variability between individuals of the same chronological age during somatic growth is great, and it is especially pronounced during puberty [14].

Furthermore, this research has verified that biological age explains in higher percentages HGS than does chronological age. When using biological age is used, HGS is greater in 5% of males and 5% of females. What this shows is that biological maturation has a significant impact on measures of muscular strength during puberty [25]. Moreover, during adolescence, the adipose tissue is predominant in girls while the muscle mass increases considerably in boys [26].

As a result, controlling for biological maturation is a powerful indictor of classification for work groups. This is especially true when dealing with variables related to physical strength, speed, and resistance [15].

In general, the findings from this study suggest that the diagnosis, classification, and/or monitoring of HGS in children and adolescents could be more precise if biological age were controlled for. However, in clinical and epidemiological practice, professionals from the health sciences tend to use international standards based on cross-sectional data and obtained based on chronological age.

In this context, based on our results, through this study, the researchers have developed normative data to assess HGS based on chronological and biological age for children and adolescents of the Maule Region of Chile. These norms could help establish thresholds for identifying levels of strength for both sexes.

Therefore, in the past few years, evaluation of HGS has gained considerable attention from researchers and health professionals. HGS is considered to be an indicator of nutritional state [27], sarcopenia, and bone fragility [28]. Furthermore, HGS serves as a way to control for different types of trauma, congenital problems, and degenerative diseases [29]. It is also used to monitor physical performance related to health [2], including providing important tans-cultural information in order to make comparisons with other regional, national, and international pediatric populations.

In general, the norms proposed in this study can be used and included in physical education programs in Chile. They may also serve as a baseline for developing comparisons over time. Their use and implementation are a reasonable alternative in terms of cost, and they may be easily administered to a large number of subjects simultaneously.

Additionally, the proposed norms serve to compare individual parameters with those of a specific population and determine if a subject falls into the appropriate category [10]. In this context, the percentiles proposed in this study establish three categories (<p15 as low, p15 to p85 as acceptable, and >p15as elevated). For example, the lower percentiles are associated with instances of weakness and/or frailness and can signal the health of children and adolescents. However, the higher percentiles are associated with better levels of strength. This demonstrates a greater participation in physical activity [18] and as a consequence, a better level of HGS performance.

Furthermore, it is necessary to point out that the data collection process was carried out in keeping with the requirements for quality control. The values for ETM inter- and intra-evaluator were less than 1.8%. This guarantees a high degree of stability when conducting the test for HGS. Moreover, the LMS method was used to create the percentiles. This allowed smoothed curves and a more effective estimation of the extremes of the percentiles [30]. However, due to the simple regression analysis, it is possible that the results obtained show a regression error. This could be interpreted as a possible inverse causality. Therefore, these findings should be interpreted with caution.

As a result, the calculations for the HGS levels for both sexes can be found using the following link: http://www.reidebihu.net/normativedh.php. This information facilitates the work of professionals working with pediatric populations since they can access in real time HGS levels according to chronological and biological age. It is also necessary to reiterate that despite the norms organized by chronological age, they are of limited use. The authors of this study provided the percentiles so that they may be used in other instances where anthropometric variables, such as weight, standing height, and sitting height, are not evaluated.

## Conclusion

In conclusion, HGS during childhood and adolescence should be analyzed and interpreted based on biological age rather than chronological age. However, in spite of this, this study, for both chronological and biological age, normative data was developed to assess the HGS of students of both sexes. These tools can assist professionals working with children and adolescents in ethnic and epidemiological contexts.
